# Understanding How Patient Experiences of Support While Attending a Weight Management Service Impacts Engagement, Dropout and Retention: A Semi‐Structured Interview Study

**DOI:** 10.1111/jhn.70159

**Published:** 2025-11-25

**Authors:** Jordan Everitt, Enzo Battista‐Dowds, Daniel Heggs, Amanda Squire

**Affiliations:** ^1^ Department of Healthcare and Food, School of Sport and Health Sciences Cardiff Metropolitan University Cardiff Wales UK; ^2^ Weight Management Service, Nutrition and Dietetics Department Aneurin Bevan University Health Board Newport Wales UK; ^3^ Department of Applied Psychology, School of Sport and Health Sciences Cardiff Metropolitan University Cardiff Wales UK

**Keywords:** attrition, dropout, obesity, retention, weight management

## Abstract

**Introduction:**

Patient retention underpins the success of weight management services, but they often face high dropout rates. This study aimed to explore patients' experiences of support during their attendance at a weight management service, and how it impacted on engagement, dropout and retention.

**Methods:**

Thirty‐seven patients from an NHS weight management service participated in semi‐structured interviews conducted either face‐to‐face or by telephone. An open‐ended question explored participants' perspectives on the need for support during weight management, aiming to understand how support affects retention. Interviews were audio‐recorded, transcribed verbatim, and analysed thematically.

**Results:**

Peer support and a positive group dynamic in weight loss interventions enhanced engagement by harnessing the value of shared experiences, helping some patients overcome feelings of isolation. Positive patient‐clinician interactions, based on understanding and free from judgment, also enhance engagement. However, clinician changes during interventions risks undermining supportive bonds. Support from family and friends provides valuable emotional and motivational support, however, the absence of such networks can exacerbate isolation. Barriers to communicating with clinicians outside of appointments can frustrate patients, in contrast to experiences of commercial weight loss groups where participants have direct access to group leaders for support.

**Conclusions:**

The support needs of patients with obesity attending weight management services vary considerably. Drawing on patient experiences can inform strategies to tailor support provision to better meet individual patient needs and enhance patient retention.

## Summary

- Clinicians influence retention by developing trust and rapport with patients and avoiding clinician changes during treatment pathways.
- Clinicians should seek to build rapport with patients built on empathy and understanding, and free from judgement, which is crucial for continued engagement for some patients.
- Effective management of group cohesion and disruption by group programme facilitators is crucial for harnessing peer support.

## 1 Introduction

In the UK, around a quarter of adults live with obesity [1]. The delivery of weight management services (WMSs) across Britain broadly follows a four‐tier pathway, with intervention intensity increasing alongside obesity severity and complexity [2]. Evaluations of WMSs show they support people to lose clinically significant sums of weight in those who complete them [3], but they are limited by high rates of attrition. Dropout rates from weight management interventions (WMIs) vary significantly but are frequently greater than 50% of participants [4].

Attrition from WMIs remains inadequately understood, and a wide range of possible factors have been reported [5]. In one meta‐analysis [6], higher adherence rates were seen from WMIs that offered social support compared to those that did not. Support can be conceptualised into two aspects: ‘functional’—the feeling of being supported, and ‘structural’—having peers available for support [7]. Social support has been incorporated into WMIs in several ways, including support buddies, social support contracts, group sessions and peer coaches [6], which suggests that, irrespective of how support is provided, ensuring patients *feel* supported during weight loss efforts may be of greatest importance.

A meta‐analysis of 13 randomised controlled trials reported better patient‐clinician relationships, through practising therapeutic techniques such as enhanced communication skills, motivational interviewing (MI), shared decision making and patient‐centred care, had a statistically significant effect on health outcomes [8]. This evidence suggests rapport between patients and clinicians may positively influence attendance and outcomes from WMIs. In professionally delivered WMIs, goal‐driven, patient‐centred counselling approaches such as MI may also harness supportive relationships [9], which in turn may contribute to the improved weight loss outcomes associated with MI [10].

Patients who prefer WMIs delivered in group settings often cite the importance of peer support [11, 12]. Wing et al [13] argued WMIs should harness the social connections of groups, and the potential benefit of this is evident from participants who cite accountability from a peer or group as an important extrinsic factor for engagement, which encourages new behaviours and helps overcome challenges [14].

The aim of this study was to develop a deeper understand of attrition in an NHS WMS. This paper reports the findings focused on patient experiences of support while attending a WMS, and how it impacted engagement, dropout and retention.

## 2 Materials and Methods

This study took place between March 2019 and October 2022 in a tier 2 NHS dietetic‐led weight management service. Service referral criteria included age ≥ 16, body mass index (BMI) ≥ 30 kg/m^2^, and a previous weight management attempt using community/commercial interventions. The WMS was available to individuals by referral from primary and secondary care health services within the Aneurin Bevan University Health Board area, in Gwent, South Wales.

The WMS has been previously described [15]. Briefly, patients attended either one‐to‐one appointments over a 6‐month timeframe, exclusively with either a dietitian, counsellor, or psychologist, or an 8‐week group programme. All appointments were conducted face‐to‐face. In addition to an intervention, patients had the option to attend one‐to‐one, 15‐min support sessions with an assistant practitioner or dietetic support worker, which could be accessed weekly, and could also be referred to the Wales National Exercise Referral Scheme.

### 2.1 Participant Recruitment

Eligibility criteria for this study included age ≥ 18 years, BMI ≥ 30 kg/m2, and opting to attend a tier 2 intervention. There were two phases to participant recruitment. For the first phase, patients who participated in a service programme to collect patient‐reported experiences via a bespoke short messaging service application provided an initial sample for recruitment [16]. Patient feedback was entered into an excel file and analysed to facilitate purposive recruitment by identifying a range of experiences, as well as reasons for continued attendance or nonattendance. Additional participants were recruited by analysing a list of missed appointments, which were cross‐referenced with the service's database to identify patients with a range of engagement levels. In total, the participant information sheet and research invitation letter were sent to 131 patients, who were given a timeframe of 2 weeks in which they could contact the research team. Following the 2‐weeks, the researcher telephoned those patients to discuss the research and participation.

### 2.2 Conducting the Interviews

This study utilised qualitative research methods to comprehensively understand attrition in an NHS WMS, and how participant experiences of support impacted retention. Semi‐structured interviews were considered the most suitable method because they can facilitate conversations centred around evidence‐informed topics, while providing participants the opportunity to discuss issues pertinent to their experiences. The full interview schedule was informed by evidence, including secondary research of attrition and adherence in WMIs [4, 5, 17, 18], in addition to patients' responses in the outlined messaging application [16]. The full interview schedule focused on five overarching themes, shown in Supporting Information S1: Material 1. This paper reports on responses to an open‐ended question asking participants to share their thoughts on the need for support during weight loss efforts, with discussion prompts including social support, support from the service, sabotage, and encouragement.

The researcher conducting the interviews was not a member of the clinical team, therefore had no prior interaction with any of the patients. Participants were offered in‐person or telephone interviews, to ease participant burden and maximise recruitment. Face‐to‐face interviews were conducted in hospitals and health centres across the health board area, and all interviews were recorded using an Olympus dictaphone. Participants gave written informed consent before face‐to‐face interviews and provided verbal (dictaphone recorded) informed consent before telephone interviews.

### 2.3 Data Analysis

Interviews were recorded digitally and transcribed verbatim into Word documents, and each transcript was read thoroughly to validate accuracy. Participants were given an identification code in consecutive order (e.g., P1 = the first participant interviewed, P2 = the second, etc), to ensure anonymisation. All transcribed documents were imported to NVivo (version 12.0), and data were analysed thematically following the six‐phase framework outlined by Braun and Clarke [19]. Firstly, all transcribed documents were read front to back, and the last page of each participant′s interview schedule was purposely left blank to note initial ideas. A systematic line‐by‐line approach was then used to generate codes using NVivo. All codes were compiled into initial theme ideas and were continuously discussed and revised amongst co‐authors, and an initial thematic map was developed to conceptualise relationships between themes. Key considerations to ensure rigour included ensuring themes were coherent and supported by the data, that themes did not encompass too much information, and that there was minimal overlap. Meetings with co‐authors were regularly held to refine the themes, ensuring each theme was distinct.

### 2.4 Ethical Approval

Ethical approval was granted by Cardiff Metropolitan University (ID 10594/PGR‐1162), and Wales REC 7 (Ref [18]/WA/1021) (IRAS: 240002).

## 3 Results

Thirty‐seven interviews were included in the analysis. Thirty‐two participants were female and five were male. The mean participant age was 49.9 (range 34–66). Fourteen participants attended the dietetic one‐to‐one intervention, 17 attended the group programme, two attended counsellor one‐to‐one, one attended psychology one‐to‐one, and three only attended the IC and did not attend an intervention appointment. Five participants attended a second intervention after the first. Characteristics of each participant are provided in Supporting Information S2: Material 2.

Seventeen interviews were conducted face‐to‐face, and 20 over the telephone. Mean interview length was 48 min (range: 20–111 min). Four overarching themes were developed, (1) support provision from the service, (2) group cohesion, (3) patient‐clinician interactions, and (4) social support networks. Sub‐themes are outlined in Table 1, and additional supporting participant quotes are provided in Supporting Information S3: Material 3.

**Table 1:** Themes and sub‐themes developed from the coded transcripts.

Theme	Subtheme
Support provision from the service	Culture of empathy
	Direct communication with the service
	Service support sessions
	Longer‐term support
Group cohesion	Peer support
	Disruptive group members
	Facilitator skill
Patient‐clinician interactions	Patient‐clinician rapport
	Clinician changes during the intervention
	Negative interactions
Social support networks	Support from family and friends
	Absence of a social support network

### 3.1 Support Provision From the Service

This theme encompasses the general provision of support from the weight management service, characterised by an embedded culture of empathy and non‐judgement, and the availability of weekly support sessions. However, some participants raised issues around access to support because of barriers in communication with clinical staff, as well as the need for longer‐term support to maintain motivation.

#### 3.1.1 Culture of Empathy

Most participants expressed that they had previously felt judged because of their weight and spoke positively about the non‐judgmental environment of the WMS. This helped foster trust and understanding and contributed to a sense of psychological safety within the service.

It was just I think being listened, not being judged, and being understood.

P29

#### 3.1.2 Direct Communication With the Service

Several participants highlighted barriers to communication with the service, as they did not have direct contact with clinical staff and were instead required to go through administrative staff at the booking centre. This undermined their access to timely support outside scheduled appointments.

It would have been nice to have a worker, where you could ring up and say you know this isn′t working, a point of call.

P11

Barriers to direct communication with service clinicians contrasted with some participants' experiences in commercial weight loss groups, where they had direct access to group leaders for motivational support.

The lady who run that class (Slimming World), she would message you during the week, to see how you are or, you'd get little cards just to say you're doing well keep it up, do you know, just little motivational things like that.

P6

#### 3.1.3 Service Support Sessions

Participants who attended the service support sessions valued them because they provided an opportunity to reflect on their efforts and gain motivational encouragement. Importantly, the support sessions were weekly and ad hoc, which meant the support was timely. Participants reported that the sessions were reassuring, as they could attend when they needed encouragement.

The (support) sessions are really important because when you have that moment that you are struggling, it helps you continue and get back on track.

P25

#### 3.1.4 Longer‐Term Support

Many participants felt that support from the service should have been available for longer, including follow‐up after interventions, due to the challenges of maintaining behaviour change and weight loss, and the fear of losing access to support.

I suppose in a way when you get to the end of it, you think, they′re not there anymore, yeah there is that feeling, I mean it would be quite nice to be able to, you know, continue with something… I did feel a little bit—heeelp (laughs), once you'd got all the way to the end.

P31

### 3.2 Group Cohesion

The dynamic between members of a group intervention influences whether participants benefit from and continue attending group sessions. Disruptive behaviour can undermine this dynamic, requiring skilful management by group facilitators.

#### 3.2.1 Peer Support

For patients who attended group sessions, peer support and shared learning facilitated better engagement by reducing feelings of isolation associated with weight management challenges.

It was sort of nice to be with other people that have got a similar sort of problem then if you like for different reasons, and um and, and to be able to talk about it.

P26

Peer interactions helped create a safe space for participants to open up to their group, discuss their feelings, and share personal challenges.

You don't want to admit some of it either, there's a lot of that, but as soon as one person does then other people feel brave enough to.

P38

Although some participants said the group programme was boring due to its academic style and lack of fun and camaraderie, this created a sombre environment that affected some participants' desire to engage.

It was very subdued, there was no laughing, no joking about, no camaraderie. It got so deep really quick, the only way to describe it is it's like being in an AA meeting, in terms of hi I′m fat and I want to lose weight, sort of, that's how it felt.

P17

#### 3.2.2 Disruptive Personalities Within the Group

Disruptive group members undermined cohesion and reduced enjoyment of the sessions for some participants. Examples included individuals who were unenthusiastic about the programme, intentionally argumentative, or exhibited dominant personalities.

The one gentleman just wanted to argue his case, I mean he wanted to argue all the time with anybody… that's why I didn't go back because I just didn′t get any benefit out of it when he kept going on.

P33

#### 3.2.3 Facilitator Skill

Participants wanted group facilitators to take greater control over disruptive group members to avert negative interactions that impacted group cohesion.

The two people in the class just have no control over it, as the meetings progressed, they (disruptive members) got more and more disruptive, more and more rude, and the two people leading it just had no control.

P12

### 3.3 Patient‐Clinician Interactions

Participant interactions with clinicians heavily influenced their engagement with the service. Positive interactions helped build rapport, trust, and understanding. However, this could be disrupted by changes in clinicians during treatment pathways. It was also important that clinicians remained mindful of patients' emotions and needs, as seemingly minor comments could unintentionally undermine motivation.

#### 3.3.1 Patient‐Clinician Rapport

Most participants wanted to develop a rapport with their clinician but described contrasting experiences. Positive rapport enabled participants to openly share their personal challenges with their clinician, which fostered a supportive bond.

I felt like I could talk to (staff member), (staff member) just made me feel at ease. I find it very difficult to open up so for it to be somebody to make me feel that comfortable, in order to talk to (staff member), is what I need.

P37

Participants who felt that rapport was lacking suggested it created a disconnect between themselves and the clinician.

You need to make people feel comfortable and try and have some understanding and, you know you need to have a rapport with people and I just felt there was none there at all.

P5

Continuity with the same clinician helped sustain rapport, while changes in clinicians during the intervention disrupted this bond. Participants expressed frustration when this led to repetitive discussions or conflicting advice, which could undermine their motivation.

You build up a rapport with somebody and its working for you and you are losing weight. I would have really liked to have stayed with the same person all of the way through.

P24

#### 3.3.2 Negative Interactions

A few participants experienced a negative interaction with their clinician, which in some instances led to participants deciding to stop attending the WMS. This mostly stemmed from comments or advice that participants disliked, leaving participants feeling like their clinician was not in‐tune with how they were feeling.

(the clinician) said, well if you look at how much weight you′ve lost in the last 9 months, there's no way you′re going to lose that in this time. They put me back by those comments… what they should have said to me was, why don't you set yourself a smaller goal and don′t put pressure on yourself.

P25

### 3.4 Social Support Networks

Several participants gained emotional support from family and friends which provided motivational encouragement. In instances where participants reported not having much social support, this could result in them feeling isolated and lacking in support, compounding their weight management challenges.

#### 3.4.1 Support from Family and Friends

Some participants said their family members also made dietary changes to help them implement the advice they were receiving, motivating their efforts to sustain behaviour changes.

(participant's daughter) helped push me and say well give it a try… every time I come home anything I had like any paperwork or anything like that she'd read and look into it, oh we'll try this, we'll do that, and it was like, as much as I was doing it, she was doing it to support me.

P27

#### 3.4.2 Absence of a Social Support Network

Absence of a social support network was perceived to hinder behavioural change efforts for some participants, due to the lack of encouragement and emotional support.

I don't have much support family wise… I don't have a lot of friends… It makes it (weight loss) harder. I find that because obviously if you have somebody that you can work along with then it does make it that much easier, someone to sort of, give you the encouragement to do it.

P15

## 4 Discussion

The aim of this study was to understand attrition in an NHS WMS by exploring how patient experiences of support impacted engagement, dropout and retention. It was evident that participants felt support from service staff, peers, and friends and family helped to facilitate engagement by positively affecting motivation and providing a source of emotional encouragement.

From the interviews, it was evident that participants felt the WMS was a safe environment free from judgement. This was important because most participants said they experience weight stigma in their lives, which could negatively impact self‐esteem. Evidence has shown that weight discrimination, including from healthcare professionals, may undermine weight loss attempts and result in poorer weight loss outcomes [20, 21]. The experience of weight discrimination can also lead to increased calorie intake [22], as well as reduced participation in physical activity [23], challenging the notion that it motivates weight loss efforts. This evidence reinforces the views expressed by participants, underpinning the need for WMSs to provide a psychologically safe space for patients.

Many participants suggested that the need for support was time‐sensitive, but the inability to directly contact clinicians outside of appointments, instead having to communicate with administrative staff at the booking service could cause frustration. This contrasted with experiences in commercial weight loss groups whereas people could communicate directly with group leaders, providing better access to timely support in the views of some participants. Providing some access to clinicians via telephone or email was considered helpful to facilitate communication between patients and clinicians according to participants from one WMI [14], which may help bridge the support needs of some patients.

Some participants wanted longer term follow‐up with the WMS to maintain support and accountability, to help them maintain behaviour changes. Weight loss is accompanied by physiological adaptations that are likely to result in weight regain because they are difficult to attenuate without effective strategies to counter them [24, 25]. Participants may also feel a sense of abandonment at the end of interventions, and services could explore the provision of longer‐term follow up, either within the service or through peer support groups [11]. An evidence review for the National Institute for Health and Care Excellence (NICE) concluded there is a need for longer‐term follow‐up after WMIs [26], which could involve a graduated exit offering ‘light‐touch’ support [27].

Participants of the current study reported that peer support during the group programme was important because it reduced feelings of isolation during weight loss efforts, and they valued shared experiences with their peers. This is consistent with previous research [11], and additionally, accountability from a peer or group can encourage new behaviours and help overcome challenges [14]. In contrast, some participants in the present study who attended the group programme reported that disruptive group members inhibited group cohesion, and they wanted facilitators to assert greater control. Both group dynamic [28], and group facilitator approach and personality influence participants' perception of a programme's success [26], and group conflict has been associated with poorer attendance, adherence, and weight loss [29]. This evidence suggests group cohesion is important for participants' continued attendance in group programmes, and factors that undermine group dynamics should be addressed by group leaders to ensure participants benefit fully from attending.

Participants value feeling listened to and understood by clinicians, consistent with previous research [11, 14]. However, clinician changes during treatment pathways undermined continuity of support and rapport according to participants in the current study. Poor patient‐provider interaction was commonly cited as a reason for dropping out of obesity treatment in Italian services [30]. Additionally, perceived lack of support from providers emerged as a core theme from interviews with participants who dropped out of the NHS Low Calorie Diet Programme Pilot [31]. In contrast, investing time to achieve high‐quality provider‐user relationships is associated with WMI success [27]. In one study, the measure of participant‐interventionist collaborative bond was a significant predictor of adherence at 6 months, which in turn predicted weight loss at 6 months [32].

Developing understanding and trust can strengthen patient‐clinician relationships, helping to prevent patients from feeling they are failing, and positivity from clinicians, even in the face of challenges, encourages continued weight loss efforts and aids patient resilience to set‐backs [33]. Clinician involvement, coupled with a broader support system helps patients to feel they have the investment of the professionals they rely upon for help [33]. However, as reported by participants in the current study, clinician changes during an intervention pathway should be avoided because it could disrupt the positive influence that clinicians can have on participant experiences.

Participants in the current study also cited the importance of social support from friends and family to maintain motivation and support behaviour change, consistent with findings from a qualitative synthesis [34]. Furthermore, support from social networks helped facilitate weight‐loss and maintenance according to participants in a study of seven focus groups, while a lack of social support created barriers [35]. In an interview study exploring weight management programme preferences, some participants even reported they would like services to incorporate family and friends in the weight loss process [36]. Participants in the present study who reported having little or no social support said the challenge of weight loss was compounded by the absence of emotional encouragement, which left them feeling isolated in their efforts. Services may benefit from countering this by exploring means of providing support to patients without social support networks.

### 4.1 Strengths and Limitations

This large qualitative study consisting of 37 interviews addresses a notable evidence gap. Qualitative studies into attrition in weight management services are scarce, particularly from UK NHS services, where engagement metrics have significant impact on the cost‐effectiveness of publicly funded services. While previous studies have often reduced participant responses to simplified lists of ‘reasons for completion/dropout,’ our research has taken a more nuanced and comprehensive approach by exploring how and why these factors influence patient engagement. This study adds detailed insight into the support needs of patients attending weight management services, and how experiences of support affect patient retention. Adding to the strengths of this study, the interviewer was not a member of the clinical team, which may have helped participants feel comfortable to express their opinions honestly.

Limitations of this study include participant demographics, as only five males participated, therefore gender differences could not be thoroughly explored. However, the proportion of men to women, the mean age of this study sample, and proportion attending each intervention were comparable with the service's broader patient characteristics [15]. The development of themes may have been determined in part by the prompts used in the interview schedule and therefore may not have been entirely inductive. However, the semi‐structured interview approach facilitated exploratory discussions in which participants were encouraged to talk about matters important to them. In addition, we did not categorise participants as ‘completers’ or ‘dropouts’ to explore differences between them. Although this was an intentional decision with the aim of taking an exploratory approach to capture a broad and comprehensive understanding of the factors which influence patient engagement, it limited the ability to describe the study sample and identify key patterns across engagement levels.

### 4.2 Recommendations

For research, further qualitative investigations into attrition are needed to explore participant experiences to improve the design, acceptability, and effectiveness of WMIs. Where possible, WMSs would benefit from collecting patient‐reported reasons for dropout, as recommended in the Star Lite core outcome measures for WMIs [37]. Studies should explore empirically evidenced variables including participant expectations and satisfaction, patient‐clinician relationships, social support, and logistical barriers, which are reported qualitatively [4, 30], but scarcely explored in studies aiming to identify factors associated with attrition.

For practice, several actionable recommendations have resulted from this study. Services may benefit from incorporating opportunities for peer support for patients with limited social support networks and those attending one‐to‐one interventions. Clinicians should seek to build rapport with patients through empathy and understanding, and free from judgement, which is crucial for continued engagement for some patients. Services might further benefit from avoiding clinician changes during treatment pathways to harness positive patient‐clinician bonds. Effective management of group cohesion and disruption by group programme facilitators is crucial. Identifying the training and development needs of group facilitators, such as motivational interviewing in groups training, or through peer supervision, may enhance management of group dynamics and cohesion, and consequently patient retention and treatment effectiveness.

## 5 Conclusion

Feeling supported during engagement with WMSs helps patients to benefit from shared experiences with their peers, feel psychologically safe in a judgement‐free environment, and help overcome feelings of isolation in the challenge of weight management in obesity. Services could help avoid risking supportive bonds by avoiding clinician changes during interventions, and in the context of group programmes, ensure facilitators effectively manage group cohesion and disruption. Understanding individual patient needs, particularly for patients who have a limited social support network, could help identify ways to facilitate greater support and encouragement.

## Author Contributions

**Jordan Everitt:** funding acquisition (equal), conceptualization (equal), methodology (equal), investigation (lead), formal analysis (lead), writing – original draft preparation (lead). **Enzo Battista‐Dowds:** funding acquisition (lead), conceptualization (equal), methodology (equal), formal analysis (supporting), writing – review and editing (equal). **Daniel Heggs:** methodology (equal), formal analysis (supporting), writing – review and editing (equal). **Amanda Squire:** funding acquisition (lead), conceptualization (equal), methodology (equal), formal analysis (supporting), writing – review and editing (equal). All authors had final approval of the submitted and published versions.

## Conflicts of Interest

The authors declare no conflicts of interest.

## Supporting information

Supporting material 1 ‐ Full interview schedule used during interviews.

Supporting material 2 ‐ Characteristics of interview participants.

Supporting material 3 ‐ Themes with additional supporting quotes.
