# Supplementary material for: Understanding How Patient Experiences of Support While Attending a Weight Management Service Impacts Engagement, Dropout and Retention: A Semi‐Structured Interview Study
Source: J Hum Nutr Diet. 2025 Nov 25;38(6):e70159. doi: 10.1111/jhn.70159 (PMC12647425; doi:10.1111/jhn.70159)
Supplement: Supplementary file 1 — Supporting material 1 ‐ Full interview schedule used during interviews. [file JHN-38-0-s002.docx]

Supplementary material 1 – Full interview schedule used during interviews

1. If you can remember, how were you referred into the service, and were you told that you were being referred to the service?

- GP
- AHP
- Nurse

1. How did you feel about the length of time between the referral and your first appointment?
2. How did you feel about the communication and information you received from the service?

- Little information provided before attending
- Slow
- Limited communication
- Inconsistent

Section 2 – weight management factors

1. What were your weight loss goals when you started with the service?
2. What were your reasons for wanting to achieve those goals?

- How did you find staying motivated?

1. What are your thoughts on the need for support during weight loss efforts?

- Social support
- Encouragement
- Sabotage
- Support from the service

1. How did you feel about your weight loss progress during your time with the service?
2. How do you feel your progress in the service compared to your previous weight loss efforts?

- Trust/believe/agree with the advice given?

1. Since leaving the service, how do you feel the service has helped you with your weight management?

- Lost weight / continued weight loss efforts
- Continued agreed dietary / exercise behaviours
- Improved understanding of portion sizes/control
- Sought extra support with weight management

1. We know that many people feel an expectation or pressure to try and manage their weight, what are your experiences and thoughts on this?

Section 3 – Times, costs & travel

1. How did you feel about the time of day and location of the appointments?
2. How did you feel about the length of time between appointments?
3. What are your thoughts on attending the service and managing your other commitments?

- Work
- Family

1. We know that many people have experienced difficulty with affording the cost of travel that’s involved with attending appointments, what were your experiences of this?
2. We know that the cost of diet and lifestyle changes can make it harder to make those changes, what were your experiences of this?

Section 4 – Health factors

1. We know that issues with mental wellbeing can affect people’s engagement with healthcare services as well as their weight management efforts, how might this have affected you during your time with the service?

- Negatively / positively affected engagement
- Stress
- Depression
- Anxiety

1. Additional physical health issues have also been shown to affect people’s engagement with healthcare services and weight management efforts, how might this have affected you during your time with the service?

Section 5 – Reflecting on overall experience with the service

1. Did the service meet your expectations?

- if not why?

1. Would you say you were satisfied with the service?

- If yes/no – what specifically?

1. If you could make any changes to the service, what changes would you make?
2. Thank you for your time and for sharing your thoughts, is there anything else you would like to add that we haven’t already discussed?
