# Supplementary material for: Understanding How Patient Experiences of Support While Attending a Weight Management Service Impacts Engagement, Dropout and Retention: A Semi‐Structured Interview Study
Source: J Hum Nutr Diet. 2025 Nov 25;38(6):e70159. doi: 10.1111/jhn.70159 (PMC12647425; doi:10.1111/jhn.70159)
Supplement: Supplementary file 2 — Supporting material 2 ‐ Characteristics of interview participants. [file JHN-38-0-s003.docx]

Supplementary material 2 – Characteristics of interview participants

| **Participant study ID** | **Sex** | **Age** | **First intervention** | **Second intervention** |
| --- | --- | --- | --- | --- |
| P1 | Male | 63 | Dietetic 1:1 | No |
| P2 | Female | 38 | Initial consultation only | No |
| P3 | *Not included in analysis as entered tier 3 service* | | | |
| P4 | Female | 50 | Dietetic 1:1 | No |
| P5 | Female | 51 | Initial consultation only | No |
| P6 | Female | 44 | Dietetic 1:1 | No |
| P7 | Female | 53 | Combined group | No |
| P8 | Female | 49 | Combined group | No |
| P9 | Female | 34 | Dietetic 1:1 | No |
| P10 | Female | 43 | Dietetic 1:1 | No |
| P11 | Female | 48 | Initial consultation only | No |
| P12 | Female | 36 | Combined group | No |
| P13 | Female | 55 | Counsellor 1:1 | No |
| P14 | Female | 54 | Dietetic 1:1 | No |
| P15 | Female | 61 | Combined Group | No |
| P16 | Male | 62 | Combined Group | No |
| P17 | Female | 40 | Combined Group | No |
| P18 | Female | 60 | Dietetic 1:1 | No |
| P19 | Male | 46 | Combined Group | No |
| P20 | Male | 66 | Combined Group | No |
| P21 | Female | 52 | Combined Group | No |
| P22 | Female | 44 | Combined Group | Dietetic 1:1 |
| P23 | Female | 45 | Combined Group | No |
| P24 | Male | 49 | Dietetic 1:1 | No |
| P25 | Female | 54 | Dietetic 1:1 | No |
| P26 | Female | 58 | Combined Group | No |
| P27 | Female | 49 | Combined Group | No |
| P28 | Female | 58 | Dietetic 1:1 | No |
| P29 | Female | 48 | Combined Group | No |
| P30 | Female | 38 | Psychology 1:1 | Combined Group |
| P31 | Female | 59 | Dietetic 1:1 | No |
| P32 | Female | 45 | Dietetic 1:1 | No |
| P33 | Female | 47 | Combined Group | No |
| P34 | Female | 58 | Combined Group | Dietetic 1:1 |
| P35 | Female | 42 | Combined Group | Counsellor 1:1 |
| P36 | Female | 54 | Dietetic 1:1 | No |
| P37 | Female | 34 | Counsellor 1:1 | No |
| P38 | Female | 58 | Dietetic 1:1 | Combined Group |
