# Supplementary material for: Understanding How Patient Experiences of Support While Attending a Weight Management Service Impacts Engagement, Dropout and Retention: A Semi‐Structured Interview Study
Source: J Hum Nutr Diet. 2025 Nov 25;38(6):e70159. doi: 10.1111/jhn.70159 (PMC12647425; doi:10.1111/jhn.70159)
Supplement: Supplementary file 3 — Supporting material 3 ‐ Themes with additional supporting quotes. [file JHN-38-0-s001.docx]

Supplementary material 3 – Themes with additional supporting quotes

| **Theme** | **Sub-theme** | **Participant quotes** |
| --- | --- | --- |
| **Support provision from the service** | Culture of empathy | **P4** “there is a slight apprehension of, maybe you’re going to be judged, they never did, they were wonderful”  **P11** “I was looking forward to being in a group of people who could support each other, and maybe get some ideas. Just people going through the same struggles, so that you can empathise with each other and you can understand”  **P27** “I got to be honest. I was pleasantly surprised how happy and helpful everyone was, you know. No one was talking down to you, all positivity”  **P3**1 “I felt the lady put me at ease really, I didn't feel that in any way, sort of talked down to”  **P33** “(The clinician) was very good, didn't make you feel uncomfortable because your weight, my weight at that time was 28 stone, didn’t make you feel uncomfortable, just offered me as much help as what I was prepared to take” |
|  | Direct communication with the service | **P11** “I had a letter to say ring up to find a course to go on, and then I had a phone call from someone to try to organise it for me, now the other ones that were starting were too far away and the times didn't work for me too to get there, what with work and children and he said, well leave it now and I'll get back in contact with you in when the next cycle was going to start and we'll get you one that’s closer to home. The next thing I had was a letter from my doctor saying that I'd been taken off the service because I didn't contact them, and at that point, I was depressed, I've asked for this help and they've taken me off of it, having contacted me. You know you lose faith in services”  **P11** “It would have been nice to have a, a worker, where you could ring up and say you know this isn't working, a point, a point of call”  **P12** “(in contrast with the weight management service) Weight Watchers you've also got the online support, so even if you can't get to a meeting you've got the app, you've got the online support that you can go into it that way”  **P13** “(in contrast with the weight management service) counsellor from, from Slimming World, you know especially the one that I had, you know she's texting, how are things going and all the rest of it, so as I say it's like somebody’s on your shoulder watching over you to say don’t eat that piece of cake don’t do that”  **P33** “I felt a little bit, hang on a minute I didn't go to the meeting so why did they not ring me? There was no follow ups to say, well you didn't come to the meeting last Wednesday is everything okay?”  **P35** “booking centre, very nice people, very professional, but they don’t have a lot of the answers sometimes, and then you're waiting on call backs from other members of staff. If you miss their call you can't call back. You’re permanently going through the booking centre”  **P35** “The weekly (support sessions) were really important that you did have an easier way of communicating with the service. If you're not engaged with that, I think it was a lot more difficult to talk to the service and if you've got a problem or you want to say look, in the last session something did actually trigger something” |
|  | Service support sessions | **P26** “again that (support sessions) was very supportive because for me I think it worked out about once a month, so it was, you know, how has your month been, so yeah I did find that very supportive”  **P31** “I find that (support sessions) very encouraging, (says support worker’s name) was, she did give me some sort of personal little tips on how to keep going and things like that. So yeah having that little time when it's sort of the shorter appointments, it was good to be able to keep in touch with someone I think”  **P35** “But then the weekly (support) sessions were really important that you did have an easier way of communicating with the service. If you're not engaged with that I think it was a lot more difficult to talk to the service and if you've got a problem or you want to say look, in the last session something did actually trigger something” |
|  | Longer-term support | **P20** “Follow up courses, be more rigid in following up these people to see how they’re doing ok to see if they’re in trouble. I think, sometimes, alright you go on a weight management course, you're giving yourself a kick up the bum. Maybe if they follow it up, they could do that as well, saying look, you know, we can help you to do this”  **P25** “I think (the programme) should be longer, because I think that to be successful and have a change in the way you view things and for it to be a permanent change, I think for anybody, it takes two years”  **P29** “I think (the programme) could have gone on for longer. I think you know, after its finished you could go and weigh, it would have been nice to have, perhaps when you go in it’s like a one to one even a fifteen-minute consultation you know, every week or every fortnight, continued support to ensure that you’re continuing to lose weight”  **P35** “I think it takes time to sink in, and I think by the time it sinks in, and you make starting changes you want to come back and reflect on it, and there is no way to come back and reflect on it really, that is something that I felt was missing. I think maybe after six months or a period of time you can go back and do a couple of sessions refresher, or you could join another group for a couple of sessions, I think either option would work. I know it’s costly and potentially keeps people into the programme a bit longer but there have been times where I felt quite alone in this”  **P37** “I would have wanted more appointments, but I can only tell you that now, having had a break from it, whereas when it ended it felt adequate, it felt enough, but now down the line I could tell you I would have wanted more, and it would have benefited me” |
| **Group cohesion** | Peer support | **P15** “it was a lovely group, there were lots of lovely people there, I did I find it a help, because they were very encouraging, all in the same boat basically”  **P22** “I just think that (the group setting) helped because you could express how you felt about, about your weight in a room full of people that were feeling exactly the same”  **P23** “I thought (the group setting) was good because you didn't feel horrible about yourself, and the group would be the same you know, they would say oh well you're not on your own, I've put this on, and everybody was pleased for everybody that lost but they were also very understanding to the people that might of put a bit on”  **P29** “knowing that you're not the only person suffering day to day with your weight and other life, there is other people of all different ages, you know male, female, suffering the same as yourself, I think having the groups knowing that you're not the only one out there” |
|  | Disruptive group members | **P12** “a lot of (group members) have very bad manners. They had no respect for the two people leading the class at all, some of them were saying I'm here because I'm told I've got to be here, I thought right well that's not going to be of any use to anybody because you’re just, you're gonna sit there, you're not going to take anything in, you’re gonna potentially be borderline disruptive, which is exactly what it was”  **P20** “there was a lot of time spent in people talking about things that were, weren’t relevant at all. There was one lady who was looking after her disabled husband, who was quite a handful apparently, and a lot of time when we talked, started talking about a certain kind of food, she’d talk about him and whether he liked it or not. So, it was diversifying away from the conversation and it was, oh here she goes again, you know so, that kind of thing, you lose track a little bit then, there was a lot of time spent in people talking about things that were, weren’t relevant at all”  **P23** “there was one gentleman and he, I know it was a serious situation, and we were doing it but at the same time there was a bit of banter going round you know. Somebody would say something, and we were laughing, he didn't appreciate that. He was like we're not here to laugh, were here to learn and I'm like right well there's no harm in having a little chuckle, that put me off a bit I was like oh I don't want grumpy people because I get sensitive to people like that” |
|  | Facilitator skill | **P16** “maybe dealing with people a bit better who were clearly not willing to give what the dietitians say a chance, or work at it”  **P20** “it was diversifying away from the conversation, and it was, oh here she goes again, you know so, that kind of thing, you lose track a little bit then, and I think the ones that were taking the course should have stepped in”  **P33** “they tried to shut (the disruptive group member) up but he never, he never, they very politely tried to keep interrupting him but no” |
| **Patient-clinician interactions** | Patient-clinician rapport | **P18** “I gelled with the lady fairly quickly, I could understand that she knew, or she understood where I was coming from. I felt comfortable with her, I didn’t feel any disapproving, I just felt comfortable, and I was able to talk to her”  **P25** “You have to build up some kind of rapport with someone in order to allow them in and talk to them, and if you do that I think it is more successful” |
|  | Clinician changes during the intervention | **P1** “that was the bit that put me off, it’s not as if you had a regular person, but it goes a bit, ohh hang on, you’re not the lady I seen last time, it might work for someone different, they don’t mind seeing a stranger every time”  **P14** “Another thing that I don’t think was a very good idea, you didn’t see the same person. I think if you could have built up a little bit of, bit more of a rapport with whoever you were seeing, because you were seeing different people you were being given different sort of advice”  **P17** “One week you’d have one girl, you’d have the girl that you are used to and then you’d have someone else. That was another thing that was wrong. It should have been kept to one person, or the two people that were originally doing it. Because you built a bond with them, then you get to know each other. If you keep chopping changing, you then got to repeat yourself over and over again, and that's frustrating”  **P24** “from my perspective about halfway through they changed dietitians, I didn’t have the same rapport with the second dietitian as I had with the first, but whether that’s just personalities not meshing, I don’t know”  **P24** “A bit disappointed (about the clinician change) initially, obviously you build up a rapport with somebody and its working for you, and you are losing weight”  **P33** “what did make me uncomfortable was that, I only went three times and on them three times we had different people talking to us, you have to go over the same things again”  **P35** “I was going to the weekly (support sessions), I only went every fortnight, when it was the same person each time that was better, because we started to get to know each other, there was a phase where every time I went there I saw a different person, and it lasted a couple of months. It was frustrating but also the sense of why am I coming here if nobody is here to be that support, if it's a case of just jumping on a scales I can do that at home” |
|  | Negative interactions | **P7** “it was a bit lecture-y you know, when I one week spoke up and said, you know, if, say like my partner or one of my children will say to me, why are you eating that chocolate bar or why are you eating that cake, you don’t, you know you don’t need that. I will then turn around and say, my immediate reaction is, don’t tell me what to eat what not to eat you know, and that would make me feel like, right I’ll have 2 now. So I did say that's my reaction to people, because I'm so fed up of people being lectured what to eat what not to eat, and I was more or less told, well that's the child in you, you know, which again I thought was a little bit demeaning to me and how I felt, I didn’t think that was really the answer”  **P13** “(the clinician) felt that, in (their) house (their partner) had to work very hard, whereas (they) could eat anything and not put any weight on, for somebody who is overweight and can walk past Greggs and put 3 pound on without even going in, yeah that (comment) is not really that helpful”  **P36** “(the clinician said) have you tried swimming? I'm really overweight, I’ve got cellulitis, varicose veins, there’s no way I'm gonna put a bathing costume on, but it was more about, well you can put, oh what are they called, tight leggings and full clothing on. I thought well that's, you know everybody is gonna look at me when I do something like that and I walk in a pool all covered up like that, so that's instant attention that I do not want” |
| **Social support networks** | Support from family and friends | **P23** “all my family supports me. always supports me my family do, my son is the biggest one, I mean today now – what you going to have for food when you come home?”  **P31** “my daughter particularly knew about that, and my mum, the ladies in the family were very supportive, I wouldn’t say too many people but my daughter, we kind of encourage each other, and I think having somebody like that is good, just to sort of give you a little nudge”  **P35** “I'm quite lucky, my best friend is quite big, and she asked me about it and I was like, I nearly cried, they nearly made me cry, and she talked me through it and she was like well you've got to go back and I had, I was lucky I had that support, but she was the only person I talked about it with” |
|  | Absence of a social support network | **P2** “because I’m a single mother, I have nobody here to turn to if I ever get upset or stressed or lonely, whatever, so that’s when I turn to food, the thing I struggle with is emotional eating because I get very lonely as a single mother, especially with a disabled child”  **P26** “my son's quite supportive, but my husband isn’t, not that he means to be like it, he's got weight issues himself, I think it's a little bit of sabotage, in a sort of kind-hearted, meaning well, he might sort of fancy chocolate, so you know he buys it for all of us, so that you know if we’re all enjoying that then obviously he doesn't feel bad about it then”  **P33** “that’s the big one for me, that's the one that makes me eat, I get no encouragement whatsoever because my husband, he’ll tell me to my face, he absolutely adores me the way I am, he doesn't want me to change, I’m not going to say it doesn’t irritate me at times, it does, because obviously I want to lose weight to be fitter for my family and my son”  “he'll (partner) sit in front of me and order a Chinese and do whatever he can so I don't lose weight, because he doesn’t want me to lose weight” |
